# Supplementary material for: Genome-Wide Association Study of Circadian Rhythmicity in 71,500 UK Biobank Participants and Polygenic Association with Mood Instability
Source: eBioMedicine. 2018 Aug 14;35:279–87. doi: 10.1016/j.ebiom.2018.08.004 (PMC6154782; doi:10.1016/j.ebiom.2018.08.004)
Supplement: Supplementary file 1 — Supplementary material [file mmc1.docx]

**Supplementary Material**

Supplementary Figure 1. Relative amplitude histogram indicating (N=2,987 Cases and N=80,352 Controls)


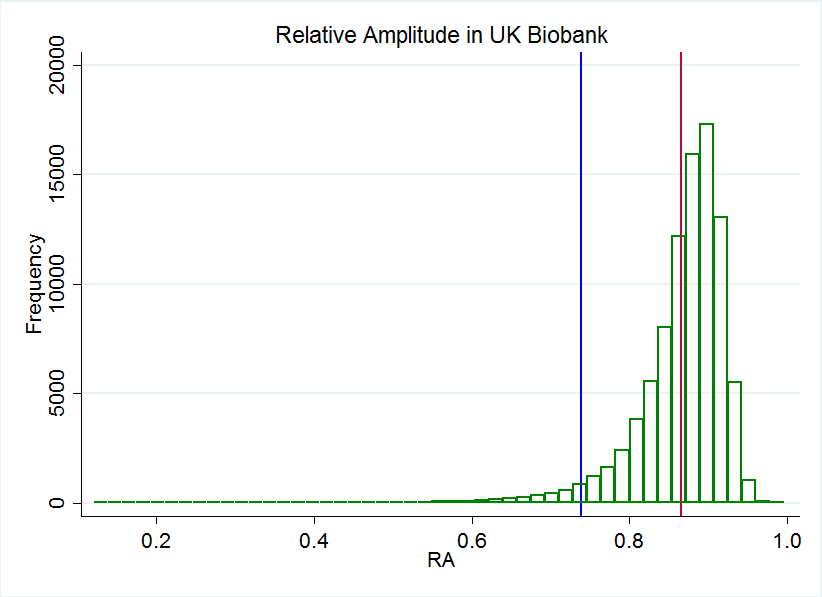


Red line represents mean value of RA. Blue line is two standard deviations from the mean, designating cases for use in the primary GWAS. These numbers indicate all (Caucasian) individuals available for GWAS before genetic exclusions and QC were applied.

Supplementary Figure 2. Representative actograms of high RA and low RA


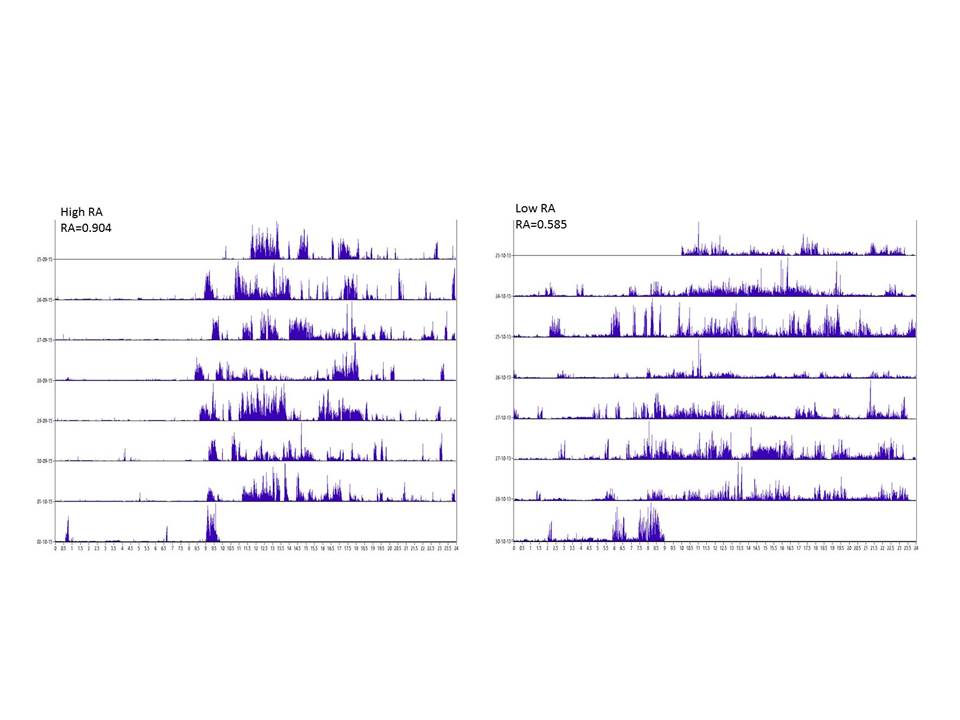


Supplementary Figure 3. Regional plots of *NFASC* and *SLC25A17*


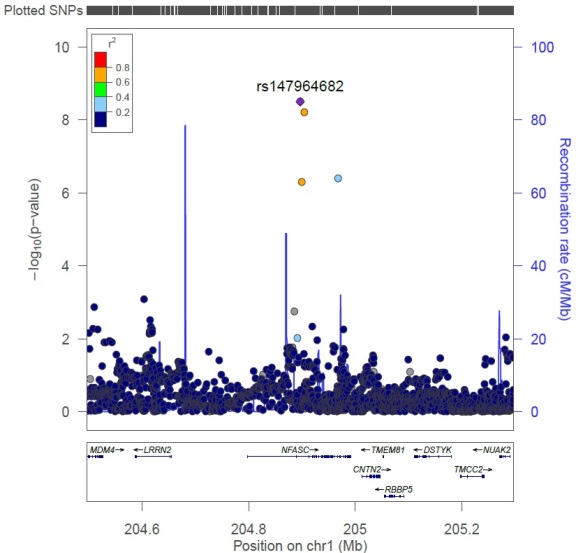

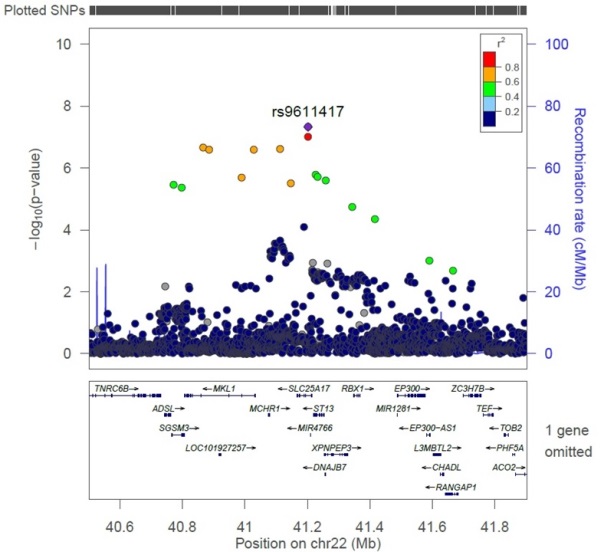


Regional plots of SNPs produced by FUMA.^35^

Supplementary Figure 4. Low RA gene-based analysis

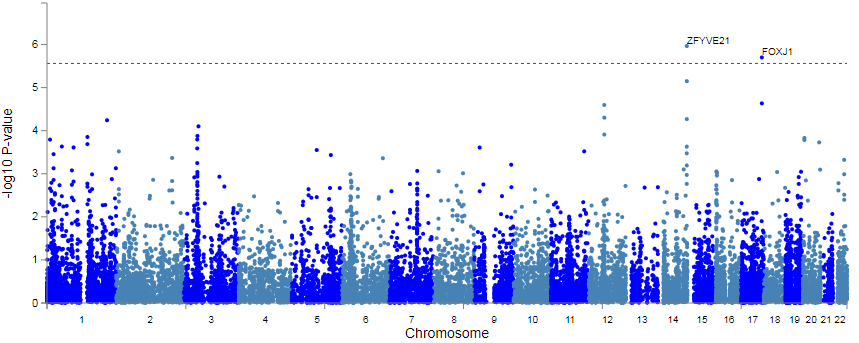


Red line represents genome-wide significance (p<5x10^-8^).

Supplementary Figure 5. Continuous RA gene-based analysis


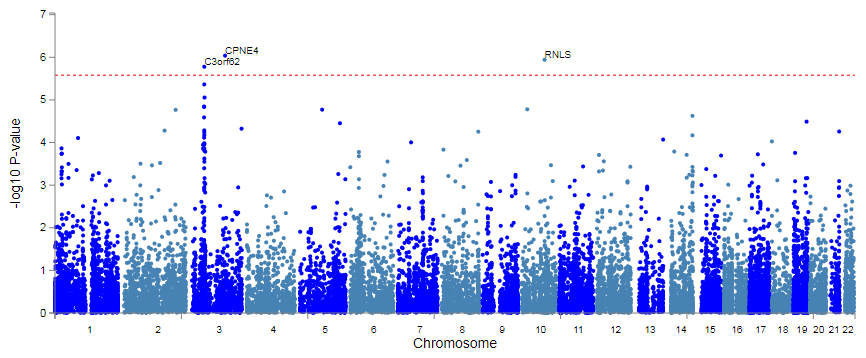


Red line represents genome-wide significance (p<5x10^-8^).

Supplementary Figure 6. eQTLs of rs9611417 box plot


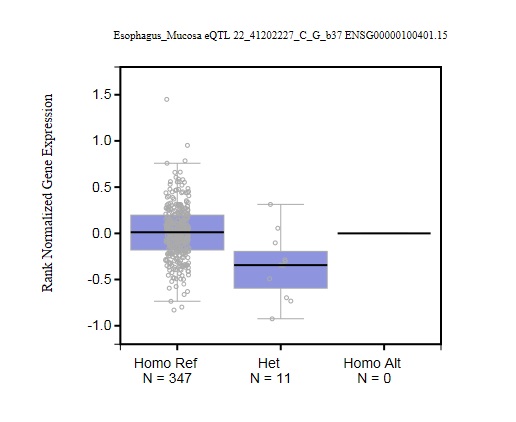

Homo Ref: rs9611417 CC, Het: rs9611417 CG, Homo Alt: rs9611417 GG. Obtained from GTex portal.^34^

Supplementary Figure 7. Tissue specific expression of *RANGAP1*


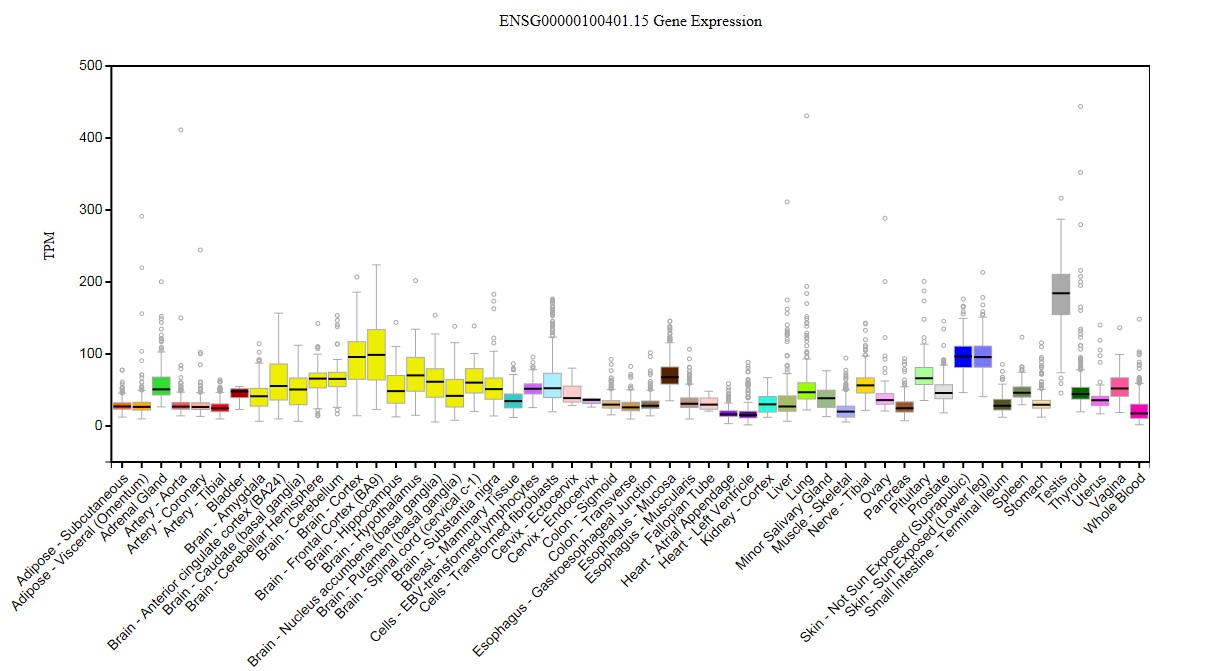


Obtained from GTex portal.^34^

Supplementary Table 1. Demographics of UK Biobank participants included in accelerometer data subset

|  | Low RA cases | Controls | Test statistic | p |
| --- | --- | --- | --- | --- |
| Age at baseline, years |  |  | 3.15 | <0·0001 |
| Mean(SD) | 55·94(0·15) | 56·39(0·03) |  |  |
| Median(IQR) | 57(50-64) | 58(52-65) |  |  |
| Age at accelerometry, years |  |  | 2·92 | <0·0001 |
| Mean(SD) | 61·69(0·15) | 62·11(0·03) |  |  |
| Median(IQR) | 62(56-69) | 63(57-69) |  |  |
| Sex |  |  | -18·18 | <0·0001 |
| Female | 1,187(39·78) | 45,405(56·57) |  |  |
| Male | 1,797(60·22) | 34,857(43·43) |  |  |
| Townsend deprivation score |  |  | -21·1 | <0·0001 |
| Mean(SD) | -0·84(0·06) | -1·91(0·01) |  |  |

Supplementary Table 2. Genome-wide significant loci associated with low RA

| SNP | Chr | Position | A1/A2 | MAF | Beta | p | Nearby gene |
| --- | --- | --- | --- | --- | --- | --- | --- |
| rs147964682 | 1 | 204,896,804 | G/C | 0·012 | 0·584 | **3**·**179x10^-9^** | *NFASC* |
| rs146042826 | 1 | 204,904,528 | A/G | 0·013 | 0·568 | **6**·**17x10^-9^** | *NFASC* |
| rs9611417 | 22 | 41,202,227 | G/C | 0·012 | 0·5616 | **4**·**753x10^-8^** | *SLC25A17* |

Supplementary Table 3. Genome-wide significant loci associated with continuous RA using BOLT-LMM

| SNP | Chr | Position | A1/A2 | Info | Beta | p | Nearby gene |
| --- | --- | --- | --- | --- | --- | --- | --- |
| rs113851554 | 2 | 66,750,564 | G/T | 0·93 | 0·0048 | **6**·**10x10^-11^** | *MEIS1* |
| 2:66782432_AC_A | 2 | 66,782,432 | AC/A | 0·92 | 0·0048 | **9**·**80x10^-10^** | *MEIS1* |
| rs11679120 | 2 | 66,785,180 | G/A | 0·93 | 0·0045 | **2**·**10x10^-8^** | *MEIS1* |
| rs115087496 | 2 | 66,793,725 | G/C | 0·92 | 0·0047 | **2**·**90x10^-9^** | *MEIS1* |
| rs142412330 | 2 | 66,802,493 | T/TCTC | 0·93 | -0·0036 | **6**·**80x10^-10^** | *MEIS1* |

Supplementary Table 4. Suggestive significant loci associated with low RA

| SNP | Chr | Position | A1/A2 | Info | Beta | p | Nearby gene |
| --- | --- | --- | --- | --- | --- | --- | --- |
| rs11715894 | 3 | 53,040,459 | A/T | 0.421 | 0.138 | 1.163E-06 | *SFMBT1* |
| rs560770640 | 3 | 53,044,907 | G/T | 0.42 | 0.136 | 1.516E-06 | *SFMBT1* |
| rs578200280 | 3 | 53,068,709 | C/T | 0.421 | 0.136 | 1.669E-06 | *SFMBT1* |
| rs151079563 | 3 | 53,194,321 | T/C | 0.447 | 0.145 | 4.014E-07 | *PRKCD* |
| rs190135744 | 4 | 32,918,842 | C/T | 0.054 | 0.285 | 2.706E-07 | *intergenic* |
| rs9277979 | 6 | 33,294,098 | T/C | 0.177 | 0.167 | 1.913E-06 | *DAXX* |
| rs546882114 | 6 | 33,308,438 | T/C | 0.181 | 0.169 | 1.949E-06 | *intergenic* |
| rs372171356 | 6 | 33,295,111 | G/A | 0.177 | 0.164 | 3.095E-06 | *DAXX* |
| rs76775274 | 7 | 91,713,047 | C/T | 0.011 | 0.521 | 1.746E-06 | *AKAP9* |
| rs117704951 | 7 | 91,876,485 | T/A | 0.011 | 0.514 | 1.921E-06 | *ANKIB1* |
| rs146314842 | 7 | 97,662,316 | T/G | 0.03 | -0.459 | 9.709E-06 | *intergenic* |
| rs289055 | 13 | 68,464,763 | G/A | 0.364 | 0.134 | 3.443E-06 | *intergenic* |
| rs289056 | 13 | 68,476,397 | T/C | 0.366 | 0.132 | 4.742E-06 | *OR7E111P* |
| rs2094932 | 13 | 68,482,537 | G/A | 0.363 | 0.13 | 8.421E-06 | *intergenic* |
| rs556389482 | 17 | 74,166,151 | A/G | 0.268 | 0.16 | 4.574E-07 | *RNF157* |
| rs562449594 | 17 | 74,171,132 | G/A | 0.264 | 0.152 | 9.400E-07 | *RNF157* |
| rs754706 | 17 | 74,150,113 | C/T | 0.264 | 0.151 | 1.026E-06 | *RNF157* |
| rs547968601 | 17 | 74,171,356 | A/G | 0.264 | 0.151 | 1.050E-06 | *RNF157* |
| rs1868822 | 17 | 74,149,524 | C/T | 0.264 | 0.151 | 1.057E-06 | *RNF157* |
| rs9277979 | 6 | 33,294,098 | T/C | 0.177 | 0.167 | 1.913E-06 | *DAXX* |
| rs546882114 | 6 | 33,308,438 | T/C | 0.181 | 0.169 | 1.949E-06 | *intergenic* |
| rs372171356 | 6 | 33,295,111 | G/A | 0.177 | 0.164 | 3.095E-06 | *DAXX* |

Supplementary Table 5. Suggestive significant loci associated with continuous RA

| SNP | Chr | Position | A1/A2 | Info | Beta | p | Nearby gene |
| --- | --- | --- | --- | --- | --- | --- | --- |
| rs10194961 | 2 | 106,304,263 | T/A | 0.338 | 0.002 | 2.80E-07 | *intergenic* |
| rs115291000 | 2 | 149,409,639 | G/A | 0.958 | 0.004 | 2.90E-06 | *EPC2* |
| rs11686221 | 2 | 107,263,240 | C/T | 0.988 | 0.006 | 4.70E-06 | *intergenic* |
| rs139169199 | 2 | 141,262,927 | C/CA | 0.96 | 0.004 | 1.10E-05 | *LRP1B* |
| rs11693221 | 2 | 66,799,986 | C/T | 0.953 | 0.004 | 1.00E-07 | *MEIS1* |
| rs142704867 | 5 | 151,700,577 | T/C | 0.964 | 0.004 | 4.40E-07 | *intergenic* |
| rs79593753 | 5 | 151,777,074 | C/T | 0.974 | 0.005 | 3.20E-06 | *NMUR2* |
| rs11538104 | 5 | 133,727,052 | T/G | 0.989 | 0.007 | 3.30E-06 | *UBE2B* |
| rs36072161 | 5 | 133,729,345 | T/A | 0.989 | 0.007 | 4.10E-06 | *intergenic* |
| rs3842139 | 6 | 34,665,678 | C/CAA | 0.264 | -0.002 | 4.10E-07 | *intergenic* |
| rs12194155 | 6 | 18,557,377 | G/T | 0.94 | -0.003 | 2.00E-06 | *MIR548A1HG* |
| rs12215669 | 6 | 18,561,460 | C/T | 0.94 | -0.003 | 2.10E-06 | *MIR548A1HG* |
| rs115595252 | 7 | 110,014,522 | T/A | 0.991 | 0.008 | 3.90E-06 | *intergenic* |
| rs7809370 | 7 | 93,708,083 | A/G | 0.63 | 0.001 | 4.80E-06 | *intergenic* |
| rs11139851 | 9 | 85,493,040 | C/G | 0.852 | 0.002 | 6.00E-07 | *intergenic* |
| rs142398474 | 11 | 18,669,128 | C/T | 0.982 | 0.006 | 1.90E-07 | *intergenic* |
| rs7951433 | 11 | 86,084,878 | C/A | 0.986 | 0.007 | 6.10E-07 | *intergenic* |
